# IgG1 Fc N-glycan galactosylation as a biomarker for immune activation

**DOI:** 10.1038/srep28207

**Published:** 2016-06-16

**Authors:** Sanne E. de Jong, Maurice H. J. Selman, Ayola A. Adegnika, Abena S. Amoah, Elly van Riet, Yvonne C. M. Kruize, John G. Raynes, Alejandro Rodriguez, Daniel Boakye, Erika von Mutius, André C. Knulst, Jon Genuneit, Philip J. Cooper, Cornelis H. Hokke, Manfred Wuhrer, Maria Yazdanbakhsh

**Affiliations:** 1 Leiden Immunoparasitology Group, Department of Parasitology, Leiden University Medical Center, Leiden, the Netherlands; 2 Center for Proteomics and Metabolomics, Leiden University Medical Center, Leiden, the Netherlands; 3 Center of Medical Research Lambaréné (CERMEL), Lambaréné, Gabon; 4 Institute for Tropical Medicine, University of Tübingen, Tübingen, Germany; 5 Department of Parasitology, Noguchi Memorial Institute for Medical Research, University of Ghana, Legon, Accra, Ghana; 6 Department of Immunology and Infection, London School of Hygiene and Tropical Medicine, London, United Kingdom; 7 Instituto de Microbiologia, Universidad San Francisco de Quito, Quito, Ecuador; 8 Dr von Hauner Children’s Hospital, Ludwig Maximilian University, Munich, Germany; 9 Comprehensive Pneumology Center Munich (CPC-M), Member of German Center for Lung Research (DZL), Munich, Germany; 10 Department of Dermatology/Allergology, University Medical Center Utrecht, Utrecht, the Netherlands; 11 Institute of Epidemiology and Medical Biometry, Ulm University, Ulm, Germany; 12 Institute of Infection and Immunity, St George’s University of London, London, United Kingdom; 13 Leiden Parasite Glycobiology Group, Department of Parasitology, Leiden University Medical Center, Leiden, the Netherlands

## Abstract

Immunoglobulin G (IgG) Fc N-glycosylation affects antibody-mediated effector functions and varies with inflammation rooted in both communicable and non-communicable diseases. Worldwide, communicable and non-communicable diseases tend to segregate geographically. Therefore, we studied whether IgG Fc N-glycosylation varies in populations with different environmental exposures in different parts of the world. IgG Fc N-glycosylation was analysed in serum/plasma of 700 school-age children from different communities of Gabon, Ghana, Ecuador, the Netherlands and Germany. IgG1 galactosylation levels were generally higher in more affluent countries and in more urban communities. High IgG1 galactosylation levels correlated with low total IgE levels, low C-reactive protein levels and low prevalence of parasitic infections. Linear mixed modelling showed that only positivity for parasitic infections was a significant predictor of reduced IgG1 galactosylation levels. That IgG1 galactosylation is a predictor of immune activation is supported by the observation that asthmatic children seemed to have reduced IgG1 galactosylation levels as well. This indicates that IgG1 galactosylation levels could be used as a biomarker for immune activation of populations, providing a valuable tool for studies examining the epidemiological transition from communicable to non-communicable diseases.

Antibodies are glycoproteins, and the N-glycans of immunoglobin G (IgG) can show considerable variation in structure, with additions of fucose, *N*-acetylglucosamine (GlcNAc), galactose and/or sialic acid to a common core (Fig. 1). Patterns in IgG Fc N-glycosylation have been found to vary with numerous physiological and pathogenic conditions, such as with age, sex, pregnancy, and certain infectious diseases, chronic inflammatory diseases, and cancers^1^,^2^,^3^,^4^,^5^. Although these associations are not well understood, it is known that certain changes in IgG glycosylation can affect antibody-mediated effector functions. It has also been shown that stimuli received during activation and differentiation of B cells could result in changes in glycosylation of the antibodies produced^6^. Furthermore, *in vivo* glycosylation changes of antigen-specific IgG1 were observed after vaccination^7^. These studies indicate that antibody glycosylation could represent a valuable readout for immunological status.

Disease patterns are changing worldwide; in affluent countries, the prevalence of communicable infectious diseases has declined, while those of non-communicable chronic inflammatory diseases have increased substantially^8^. Developing countries are still at the forefront of this transition, with large differences in prevalences of communicable and non-communicable diseases in rural and urban areas. The mechanisms underlying these diseases may be rather different. Infections are often associated with strong inflammatory responses resulting from activation of immune cells by pathogen-associated molecular patterns and foreign antigens^9^. At the same time, many non-communicable diseases that are seen in affluent countries or upon urbanisation are also associated with tissue inflammation. These diseases might result from triggering of the immune system by a different array of stimuli such as damage-associated molecular patterns, self-antigens, and/or allergens^10^.

This raises the question how IgG glycosylation patterns differ with infection pressure and with affluence and urbanisation. It is possible that IgG glycosylation patterns could provide a biomarker of immune activation in the context of urbanisation, which is a much needed tool for epidemiological studies.

## Results

### Study populations

The study was conducted among 700 school-aged children from urban and rural communities in Gabon, Ghana, Ecuador, the Netherlands and Germany. The study groups showed considerable heterogeneity in factors such as weight, height, prevalence of parasitic infections and urbanisation (Table 1).

### IgG glycosylation pattern

IgG1 galactosylation levels in children appeared to follow a developing country-affluent country gradient and a rural-urban gradient, with higher levels of IgG1 galactosylation in children from more affluent countries and from more urban communities (Fig. 2a). Thus, IgG1 galactosylation levels were higher in children from the high-income countries Germany and the Netherlands than in children from the lower-income countries Gabon, Ghana and Ecuador. Furthermore, in countries where rural to urban communities could be studied (Gabon, Ghana and Germany), IgG1 galactosylation levels were higher in urban as compared to rural children (Supplementary Table S1-2). In Ghana, the galactosylation levels were not only compared between urban and rural children, but also amongst urban children: a group of children was from a school that served high socioeconomic status families, while another group was from a school that served low socioeconomic families. The galactosylation levels were highest in children from UP (University of Ghana Primary School Legon) (urban, high socioeconomic status) and lowest in children from rural Ayikai Doblo (AD). Children from rural Mayera (MA) and from Jamestown (JT) (urban, low socioeconomic status) had intermediate levels.

Regarding other forms of glycosylation, the gradient for IgG1 sialylation largely followed the gradient for IgG1 galactosylation when comparing communities within countries, with higher sialylation levels in more urban communities (Supplementary Fig. S1A). This association is expected, as sialic acid is added to galactose, and therefore, galactosylation is required for sialylation. However, when considering the number of sialic acids per galactose moiety (SA/Gal), the order of groups was roughly reversed, with a higher SA/Gal ratio in more rural communities (Supplementary Fig. S1B). A rural-urban gradient could be present for IgG1 fucosylation, as fucosylation levels were relatively low in the rural Ghanaian communities and both Gabonese communities (Supplementary Fig. S1C). There was no clear pattern for bisecting GlcNAc (Supplementary Fig. S1D), as is also reflected by an absent correlation with galactosylation (Supplementary Table S4).

Glycosylation patterns of IgG2 and IgG4 correlated with those of IgG1 (Supplementary Fig. S2, Supplementary Table S5). However, IgG4 levels were too low in many samples from the Netherlands and Germany to allow analysis of glycosylation patterns.

### Correlation between IgG1 galactosylation and markers of inflammation and immune activation

The rural-urban gradient of IgG1 galactosylation was further studied in relation to the acute-phase C-reactive protein (CRP) as a marker of inflammation. Overall, CRP levels, amongst the studied communities, were in the reverse order of IgG1 galactosylation levels (Fig. 2b). Children from Germany and the Netherlands had the highest levels of IgG1 galactosylation and the lowest levels of CRP, while children from Gabon and Ghana had the lowest levels of IgG1 galactosylation and the highest levels of CRP. A significant, albeit weak, negative correlation was found between IgG1 galactosylation and CRP levels (Supplementary Fig. S3A).

A negative correlation was also found between IgG1 galactosylation and total IgE levels, which is associated with activation of the immune system as seen with allergy and parasitic infections (Supplementary Fig. S3B)^11^. Children from Germany and the Netherlands had the lowest levels of total IgE and highest levels of IgG1 galactosylation. Communities within countries followed a rural-urban pattern with high levels of IgE in rural communities and low levels in urban communities (Fig. 2c).

### IgG1 galactosylation can be predicted by parasitic infections

Samples from Ecuador provided the opportunity to disentangle the relative contribution of infections or other factors associated with urbanisation to IgG1 galactosylation levels. In Ecuador, all of the 7 communities studied were rural, but within the rural communities the degree of urbanisation of the communities was assessed by measuring infrastructure, socioeconomics, and lifestyle at community level (definitions in Supplementary Table S6)^12^. A low galactosylation level was significantly correlated with (a) the presence of a telephone system, and high proportions of (b) soil-transmitted helminth (STH) infections, (c) households with a pet dog, and (d) crowded households (Fig. 3a).

As parasite prevalence data, not only for STH (encompassing hookworm, roundworm, whipworm and/or threadworm) but also schistosomiasis and malaria, were available for Ghanaian and Gabonese children as well, correlations between parasitic infections and IgG1 galactosylation could be assessed beyond Ecuador. In Ghana, positivity for malaria, schistosomiasis or STHs was negatively correlated with IgG1 galactosylation, but although significant, this was a weak correlation (Fig. 3b). In Gabon, a moderate negative correlation with positivity for schistosomiasis was found, but no correlation with malaria or STHs, which might be due to low statistical power. Taken together, parasitic infections are correlated with lower levels of IgG1 galactosylation in Ecuador, in Ghana, and in Gabon. Therefore, the differences in IgG1 galactosylation between countries and communities could be driven by immune activation due to infectious agents, such as micro-organisms and parasites.

To further investigate this hypothesis, linear mixed (multi-level) modelling was used to describe IgG1 galactosylation for all communities from all countries, as shown in Table 2. This modelling approach allowed clustering of communities, thereby taking community effects into account (irrespective of country). STH infection was a significant negative predictor of IgG1 galactosylation, and schistosomiasis and CRP level near-significant predictors. IgE level was not a significant predictor, as the variation was already explained for by parasitic infections. Age and sex were irrelevant predictors for this study population, likely because of the low and small age range. Thus, this modelling approach shows that parasitic infections, which are strong inducers of immune activation, could explain differences in IgG1 galactosylation.

### IgG1 galactosylation and immune activation

To assess whether the association between IgG1 galactosylation and parasitic infections can be generalised to immune activation, we analysed samples from subjects from a rural and urban community in Germany classified as being asthmatic or not. In both areas, the asthmatic children had higher levels of total IgE than healthy children (Fig. 4). In line with the concept that immune activation in general appears to be associated with lower IgG1 galactosylation, in both communities, children with asthma showed a tendency for lower levels of IgG1 galactosylation than healthy controls.

## Discussion

Glycosylation of antibodies is known to vary with numerous physiological and pathological conditions. This study shows that galactosylation of IgG1 Fc N-glycans varies considerably between affluent and developing countries and also between urban and rural communities in the same country, where lower galactosylation levels are seen in less affluent countries and in more rural communities. Regarding factors that could explain this pattern, when considering all study participants, a negative correlation was found between CRP levels, a marker of inflammation, and IgG1 galactosylation levels. Moreover, total IgE levels, a marker of immune activation associated with parasitic helminth infections and allergy, were also negatively associated with IgG1 galactosylation levels. Although rural-urban differences could be explained by exposure to microorganisms and parasites, lifestyle and environmental difference could also play a role. We had data available from Ecuadorian rural communities, where information on infrastructure, socioeconomic and lifestyle measures was available in addition to parasitic infections. The data supported the contribution of infections, as IgG1 galactosylation was inversely correlated with the percentage of STH infections, pet dogs, and household crowding, but not with other factors related to urbanisation such as housing characteristics or education level of the parents. Parasitic infections, not only STHs but also schistosomiasis and malaria, were also found to be inversely correlated with galactosylation among Ghanaian and Gabonese children. Finally, linear mixed (multi-level) modelling of all communities from all countries in a single model confirmed that parasitic infections explained the IgG1 galactosylation pattern best.

Although decreased IgG1 galactosylation was best explained by parasitic infections in our study, this could perhaps be generalised to immune activation. During life, people are continuously challenged with antigens, and the number of experienced immune activations will increase with age. Thus, the hypothesis that low levels of IgG1 galactosylation are associated with immune activation is supported by the notion of decreased IgG1 galactosylation levels with increasing age^2^,^13^. Also acute systemic inflammation and chronic inflammatory conditions have been shown to be associated with glycosylation changes^14^,^15^,^16^. In line with this, our study shows a similar pattern where children with asthma tend to have lower IgG galactosylation levels as well. Given the rural-urban pattern of IgG galactosylation levels, the pattern seen with CRP, total IgE, parasitic infections and children with and without asthma, we propose that reduced IgG1 galactosylation is a biomarker of immune activation.

The mechanism behind altered IgG glycosylation is not fully understood. With respect to galactosylation, one possibility is that, in the face of increasing amounts of IgG1 produced by a single plasma B cell, an insufficient capacity of galactosyltransferases leads to lower galactosylation. However, it is known that upon vaccination both galactosylation of antigen-specific IgG1 and antibody titres increase^7^, while for rheumatoid arthritis IgG titres and galactosylation are not associated^4^. Therefore, the IgG galactosylation level differences are probably not simply a by-product of hyperactive antibody production.

Another possibility is that IgG galactosylation changes are induced by (micro-)environmental factors that influence B cell activation. Such factors could reduce the activity of enzymes such as glycosyltransferase^17^, or favour expansion of B cell populations producing antibodies with specific glycosylation profiles^6^,^18^. A study by Wang *et al.* has shown that CpG oligodeoxynucleotide, interleukin-21, and interferon-γ increase galactosylation and CpG oligodeoxynucleotide and interleukin-21 increase sialylation, while all-trans retinoic acid (a natural metabolite of vitamin A) decreases galactosylation and sialylation levels^6^. Therefore, different environmental exposures such as pathogens might be responsible for the lower levels of galactosylation in our rural study participants.

In terms of functional consequences, low IgG galactosylation levels are associated with several inflammatory diseases, such as rheumatoid arthritis, juvenile onset chronic arthritis, systemic lupus erythematosus, multiple sclerosis, Crohn’s disease, tuberculosis and leishmaniasis^4^,^19^,^20^,^21^,^22^,^23^. In contrast, increased galactosylation is seen during pregnancy, and in rheumatoid arthritis patients who experience pregnancy-induced remission^5^,^24^. This suggests that increased galactosylation of antibodies might be functionally more anti-inflammatory^25^. Karsten *et al.* confirmed this anti-inflammatory property by showing in mice that high galactosylation of IgG immune complexes promotes the association of FcγRIIB and dectin-1, which blocks the pro-inflammatory effector functions of C5aR and CXCR2^26^. Therefore, this would indicate that people with higher levels of immune activation would have more pro-inflammatory antibodies, as they have lower levels of IgG galactosylation.

In our study, the developing country-affluent country pattern and rural-urban pattern due to immune activation was most clear for IgG1 galactosylation, but was also seen for IgG2 and IgG4 subclasses. Furthermore, next to galactosylation, IgG sialylation and fucosylation were reduced for rural children. Although controversial for sialylation, both modifications might have anti-inflammatory effects. Sialylation was found to contribute to the beneficial effects of intravenous immunoglobulin (IVIg) treatment in some studies^27^,^28^, and afucosylated IgG1 was found to be a potent inducer of antibody-dependent cell-mediated cytotoxicity^29^,^30^,^31^. While the highest levels of sialylation were found in affluent countries and more urban communities, the number of sialic acids per galactose moiety (SA/Gal) were found to be highest in the more rural communities. This indicates separate regulation of sialylation and galactosylation, leading to smaller differences in sialylation between populations than for galactosylation. Nevertheless, the overall reduced sialylation and fucosylation in rural children could strengthen the suggested pro-inflammatory effects of reduced galactosylation.

It could be speculated that IgG glycosylation adds another layer of control to avoid antibody-induced pathology. Circulating antibodies could be kept in an anti-inflammatory state by certain glycosylation patterns, but upon danger and with the proper set of signals, more effective antibodies could be produced by switching to a more pro-inflammatory glycosylation profile. Therefore, the pro-inflammatory IgG glycosylation profile found in rural children might be better in fighting the higher infection pressure in the rural environment. In affluent countries, where infectious pressure is reduced, antibodies remain in their anti-inflammatory circulating state, with high levels of galactosylation. However, the switch to pro-inflammatory glycosylation might be aberrant in the case of autoimmune diseases, making IgG glycosylation a target for studies of disease mechanisms and therapeutics.

An interesting aspect of the degree of IgG1 galactosylation could be its use as a biomarker for immune activation. This study is one of the first comparing IgG glycosylation in various human populations, and shows the IgG1 galactosylation pattern amongst various rural and urban populations. We have used plasma and serum samples collected with different protocols and stored for different periods of time, however, the same galactosylation pattern is seen in each of the studied countries: lower galactosylation in communities where immune activation is higher due to higher exposure to microorganisms and parasites. Therefore, IgG galactosylation proves to be very stable, which is a prerequisite for a good biomarker. This also shows that, although genetics could explain some variation between countries, environmental factors have a strong effect^32^. Furthermore, IgG1 galactosylation, as a biomarker for immune activation, could be a valuable tool in studies examining infection pressure in developing countries or the prevalence of non-communicable inflammatory diseases in affluent regions of the world.

## Methods

### Study populations

School-aged children from Ecuador, Gabon, Germany, Ghana and the Netherlands were included in this study. Plasma or serum samples were obtained as part of other studies as described before^12^,^33^,^34^,^35^,^36^,^37^,^38^, and in accordance with guidelines approved by local medical ethical committees (Hospital Pedro Vicente Maldonado, Pichincha Province, Ecuador; International Foundation of the Albert Schweitzer Hospital, Lambaréné, Gabon; University of Münster, Münster, Germany; Ulm University, Ulm, Germany; Noguchi Memorial Institute for Medical Research Institutional Review Board, Accra, Ghana; Utrecht University Medical Center, Utrecht, the Netherlands) and written or thumb-printed informed consent of parents or legal guardians of the children. 193 plasma and serum samples came from a study conducted amongst rural Afro-Ecuadorian communities in Esmeraldas province, Ecuador, in 2005–2007^12^. 39 samples were from a study conducted in the rural community of PK15 (PK) and semi-urban community of Lambaréné (LA) in Gabon in February 2005^33^,^34^. 49 samples were from a study conducted in 1995–1996 in urban Munich, southern Germany^37^. 149 samples were from a study in 2007 in rural areas around Ulm, southern Germany^35^. 323 serum samples came from a study conducted in Ghana, in two rural communities (AD and MA), an urban community with low socio-economic status (JT) and an urban community with high socio-economic status (UP), in 2003^38^. 20 samples from the area of Utrecht in the Netherlands were collected in 2008–2009^36^.

Gabonese and Ghanaian children were tested for STH infection, schistosomiasis and malaria. Ecuadorian children were tested for STHs only; schistosomiasis is not endemic. In Ghana, single samples were used for parasitology^38^. 25 mg stool samples were used for the detection of hookworm, *Ascaris lumbricoides* (roundworm) and *Trichuris trichiura* (whipworm) by Kato-Katz method. 10 mL urine was filtered through a 10-mm-pore filter before detection of *Schistosoma haematobium* eggs by microscopy. Giemsa-stained thick-blood smears were used to detect malaria parasites by microscopy. In Gabon, stool samples were tested 1 or 2 times for *A. lumbricoides* and *T. trichiura* by Kato-Katz method^33^,^34^. Urine samples were tested 3 times for *S. haematobium* and an additional staining with ninhydrin solution was used. Malaria was determined by a single Giemsa-stained thick-blood smear. In Ecuador, single stool samples were used to detect hookworm, *A. lumbricoides*, *T. trichiura*, and *Strongyloides stercoralis* (threadworm) by modified Kato-Katz and formol–ether concentration methods^39^. German and Dutch children were not tested, as Germany and the Netherlands are not endemic for any of these parasitic infections.

Parent-reported asthma symptoms were known for German and Dutch children. Negative children were included in the main analysis; asthmatic children were only included for the comparison with healthy controls. No asthma data was available for the children from Ecuador, Gabon and Ghana. Body weight and height were determined for most children, from which age-standardised z-scores for body mass index (BMI) were calculated according the World Health Organisation Reference^40^. Furthermore, the communities in Ecuador have been extensively characterised by Rodriguez *et al.* to study the relationship between urbanisation and the prevalence of wheeze (Supplementary Table S6)^12^.

### IgG glycosylation analysis

The glycosylation of IgG1, IgG2 and IgG4 was analysed in June and July 2012 as described previously^7^,^41^. Briefly, the antibodies were captured from plasma/serum by affinity chromatography using Protein A beads. After tryptic digestion, the samples were analysed by fast nano reverse phase (RP) liquid chromatography (LC) electrospray ionization (ESI) quadrupole time-of-flight (Q-TOF) mass spectrometry (MS). The datasets were internally calibrated using a list of known glycopeptides^41^ and processed with Bruker DataAnalysis 4.0, MS Align2, MZmine, Xtractor2D and Microsoft Excel 2010 software. Detected glycopeptides are listed in Supplementary Table S7.

**Figure 1:**
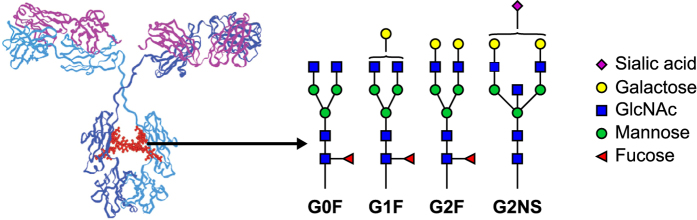
IgG1 with heavy chains in blue, light chains in purple and glycans, attached to Asn-297 of both Fc chains, in red. Examples of IgG Fc N-glycan structures are shown on the right. They can differ by additions of galactose (G), fucose (F), bisecting *N*-acetylglucosamine (GlcNAc, N) and/or sialic acid (S). The left figure was adapted from Arnold *et al.*^3^ and reproduced with permission of Prof. Dwek and Annual Reviews in the format Republish in a journal/magazine via Copyright Clearance Center. Glycan structures were drawn with GlycoWorkbench^44^.

**Figure 2:**
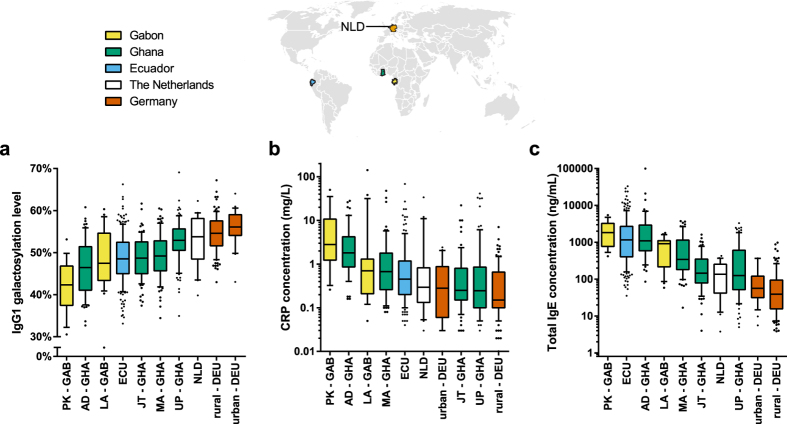
(**a**) IgG1 galactosylation, (**b**) CRP levels and (**c**) total IgE levels compared between various communities and countries. Boxplots have 10–90% whiskers. The world map was based on a figure from Wikimedia Commons and modified using Adobe Illustrator CC 2014 (Adobe Systems Inc., San Jose, CA, USA).

**Figure 3:**
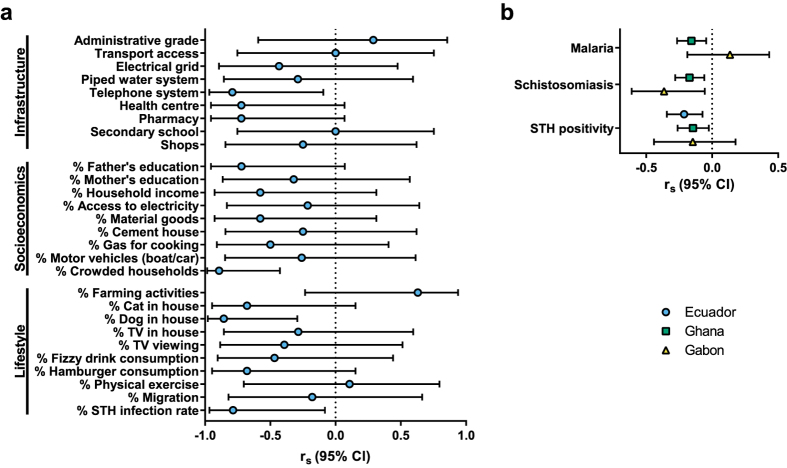
(**a**) Spearman’s correlation between median IgG1 galactosylation and factors associated with infrastructure, socioeconomics and lifestyle amongst Ecuadorian communities. Definitions of the variables are listed in Supplementary Table S6. Correlations are based on community data (n = 7). (**b**) Spearman’s correlation between IgG1 galactosylation and parasite infection status per individual, in Ecuador, Ghana and Gabon. Shown are Spearman’s rho correlation coefficient (r_s_) values with corresponding 95% confidence intervals.

**Figure 4:**
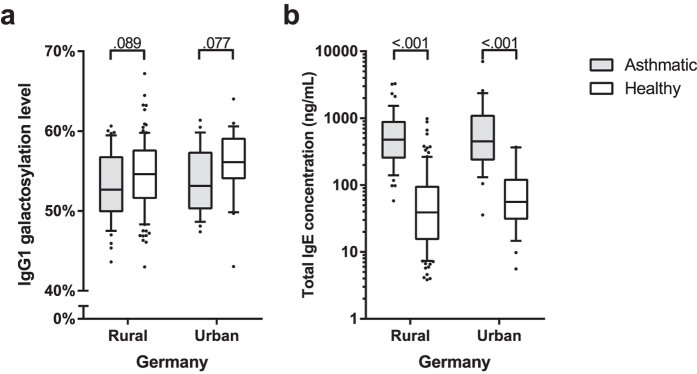
(**a**) IgG1 galactosylation and (**b**) total IgE levels in rural and urban German children, with and without asthma. P values as determined by Mann-Witney U tests are indicated. Boxplots have 10–90% whiskers.

To obtain the relative intensities of a total of 50 glycopeptide species from IgG1, IgG2, and IgG4, three isotopic peaks were integrated, summed, and normalised to the total subclass specific glycopeptide intensities. Subsequently, the level of galactosylation was calculated for IgG1 and IgG2 using the formula 0.5 × (G1F + G1FN + G1FS + G1FNS + G1 + G1N + G1S + G1NS) + G2F + G2FN + G2FS + G2FNS + G2 + G2N + G2S + G2NS, and for IgG4 using 0.5 × (G1F + G1FN + G1FS + G1FNS) + G2F + G2FN + G2FS + G2FNS. In this formula, G is short for galactose, and is followed by a number indicating how many galactoses are present in the glycan (Fig. 1). The presence of fucose is indicated by F, of bisecting GlcNAc by N, and of sialic acid (*N*-acetylneuraminic acid) by S. The level of sialylation was calculated for IgG1 and IgG2 by G1FS + G2FS + G1FNS + G2FNS + G1S + G2S + G1NS + G2NS and for IgG4 by G1FS + G2FS + G1FNS + G2FNS. The number of sialic acids per galactose moiety (SA/Gal) was determined by the level of IgG sialylation divided by 2× the level of galactosylation. The level of fucosylation was determined by G0F + G1F + G2F + G0FN + G1FN + G2FN + G1FS + G2FS + G1FNS + G2FNS for IgG1 and IgG2 only, as afucosylated species of IgG4 remained below detection limit. The incidence of bisecting GlcNAc was calculated for IgG1 and IgG2 by G0FN + G1FN + G2FN + G1FNS + G2FNS + G0N + G1N + G2N + G1NS + G2NS and for IgG4 by G0FN + G1FN + G2FN + G1FNS + G2FNS.

### C-reactive protein analysis

Plasma/serum CRP concentrations were measured in May 2013 as described before^42^ by sandwich enzyme-linked immunosorbent assay (ELISA) using 4000× diluted anti-human CRP IgG (The Binding Site, Birmingham, UK), 1000× diluted horseradish peroxidase-conjugated anti-human CRP IgG (AbD Serotec, Kidlington, UK), and the World Health Organisation (WHO) 1st international standard of human CRP (National Institute for Biological Standards and Control (NIBSC), Potters Bar, UK). Plasma/serum samples were diluted 200× (out of range samples were repeated diluted 100× or 2000×). Substrate solution was 0.1 mg/mL 3,3′,5,5′-tetramethylbenzidine in citrate/phosphate buffer, pH 4.5. Plates were analysed by absorbance at 450 nm with a reference wavelength of 490 nm using a Dynal plate reader (DYNEX Technologies, Worthing, UK).

### Total IgE analysis

Plasma/serum total IgE concentrations were measured in March 2014 by ELISA. Maxisorp plates (Nunc, Roskilde, Denmark) were coated overnight with shaking with 1000× diluted polyclonal rabbit anti-human IgE capture antibody (Dako, Glostrup, Denmark) in 0.1 M sodium bicarbonate buffer, pH 9.6, at 4 °C, and blocked with shaking for 1 h with 2% bovine serum albumin fraction V (Roche, Mannheim, Germany)/phosphate buffered saline at room temperature. Plates were then incubated with shaking for 1 h with 100 μL of 20× and 200× diluted plasma/serum samples and control samples at room temperature. The WHO 3rd international standard of human serum IgE (NIBSC) was applied at a 10-step 2× serial dilution starting at 200 ng/mL. After washing, the plates were incubated with shaking for 1 h with 1000× diluted biotinylated goat anti-human IgE, ε-chain specific, detection antibody (Vector Laboratories, Burlingame, CA, USA) followed by 3000× diluted streptavidin-alkaline phosphatase conjugate (Roche) for 1 h at room temperature, and by 100 μL 1 mg/mL p-nitrophenylphosphate substrate (Roche) in 0.1 M diethylanolamine buffer for 25 min. The reaction was stopped by 100 μL 3 M NaOH and absorbance was measured at 405 nm. The detection limit was 4.0 ng/mL.

### Statistical analysis

Data analysis was performed using IBM SPSS Statistics version 20 for Windows (IBM Corp., Armonk, NY, USA). Graphs were made using GraphPad Prism version 6 for Windows (GraphPad Software, San Diego, CA, USA). CRP and total IgE levels were log_10_ transformed for statistical analyses. Spearman’s correlation was used to assess various associations with IgG galactosylation at the level of individuals, except for correlations with infrastructure, socioeconomic and lifestyle factors in Ecuador, which were performed at the community level. Comparisons of IgG1 galactosylation between countries and communities were performed with the non-parametric Kruskal-Wallis H test, followed by Dunn-Bonferroni post-hoc test, or Mann Whitney U test. Random-intercept fixed-slope linear mixed (multi-level) modelling was used to describe the glycosylation differences considering all communities, irrespective of country. Linear mixed models are extensions of linear regression, where fixed effects are comparable to conventional linear regression and random effects are introduced to take into account cluster effects, in this case the various communities, by estimating an intercept for each community. The starting model contained age, sex, CRP level, total IgE level, schistosomiasis status and STH infection status as fixed effects. Ecuadorian children were assumed negative for schistosomiasis. Dutch and German children were assumed negative for schistosomiasis and STH infection. The variance component for communities was significant according to the associated likelihood ratio test (P < 0.001). Therefore, communities were kept in the model as a random effect. Subsequently, a top-down modelling strategy was used as described by West *et al.*^43^ to remove non-significant fixed factors and to reach the final model as shown in Table 2. (Two-tailed) P values below .05 were considered statistically significant.

## Additional Information

**How to cite this article**: de Jong, S. E. *et al.* IgG1 Fc N-glycan galactosylation as a biomarker for immune activation. *Sci. Rep.* **6**, 28207; doi: 10.1038/srep28207 (2016).

## Supplementary Material

Supplementary Information

## Figures and Tables

**Table 1:** Characteristics of the study populations.

Population			n	Age (range)	Male sex	Weight	zBMI^a^	Schistosomiasis	STH	Malaria
GAB	PK	Rural	16	9.0 (7–11)	33.3%	22		100.0%	87.5%	37.5%
	LA	Semi-urban	23	8.0 (7–12)	34.8%	28		17.4%	13.0%	13.0%
GHA	AD	Rural	68	10.0 (5–14)	45.6%	25	−0.68	50.8%	33.8%	58.3%
	MA	Rural	87	10.0 (5–14)	55.2%	27	−0.80	30.1%	19.7%	44.9%
	JT	Urban, low SES	87	10.0 (6–17)	66.3%	27	−1.16	0.0%	17.1%	0.0%
	UP	Urban, high SES	81	9.0 (5–13)	53.2%	43^b^	0.65^b^	0.0%	3.4%	6.7%
ECU	TA	Rural	31	9.0 (8–12)	51.6%	26.6	−0.43		93.5%	
	LP	Rural	30	10.5 (8–12)	36.7%	28.6	−0.08		66.7%	
	SA	Rural	35	10.0 (8–12)	57.1%	29.0	−0.60		68.6%	
	BZ	Rural	24	10.0 (8–12)	58.3%	28.6	0.23		50.0%	
	ZG	Rural	32	11.0 (8–12)	50.0%	31.6	−0.14		71.9%	
	TR	Rural	18	11.0 (8–12)	66.7%	30.1	−0.48		27.8%	
	PO	Rural	23	9.0 (8–12)	56.5%	27.0	−1.91		4.3%	
NLD	Urban and semi-urban	20	9.5(8–12)	60.0%						
DEU	Healthy	Rural	100	8.0 (6–11)	40.0%	30.1	0.45			
		Urban	25	9.0 (9–10)	60.0%	39.0^b^	0.06^b^			
	Asthmatic^c^	Rural	49	9.0 (6–11)	61.2%	29.6	0.21			
		Urban	24	9.0 (9–10)	66.7%	33.3^b^	0.09^b^			

Values represent medians (min-max), percentage male or prevalence. Age is shown in years and weight in kg.

^a^Age-standardised z-scores for body mass index (BMI) according to the WHO Reference^40^.

^b^Information was available for <50% of participants.

^c^Asthmatic children were only included in the comparison of healthy and asthmatic Germans as in Fig. 4. For the other analyses, only healthy Germans were included. STH = soil-transmitted helminth infected, GAB = Gabon, GHA = Ghana, ECU = Ecuador, DEU = Germany, NLD = the Netherlands, SES = socioeconomic status.

**Table 2:** The starting linear mixed (multi-level) model with IgG1 galactosylation as dependent variable is shown, as well as the final model after sequential removal of total IgE level, age, sex, CRP level, and schistosomiasis status as non-significant fixed effects.

Model	Effects	Parameter	b	P value	95% CI	
Starting model	Fixed	Intercept	47.64	<0.001	43.44	51.84
		Sex	0.68	0.155	−0.26	1.61
		Age	−0.06	0.670	−0.33	0.21
		Log(CRP level)	−0.66	0.068	−1.37	0.05
		Log(Total IgE level)	−0.11	0.787	−0.92	0.69
		Schistosomiasis	−2.41	0.024	−4.50	−0.32
		STH infection	−1.24	0.059	−2.53	0.05
	Random	Residual	30.29	<0.001	26.88	34.15
		Intercept (Community)	5.55	0.052	2.02	15.21
Final model	Fixed	Intercept	48.62	<0.001	46.82	50.43
		STH infection	−1.37	0.028	−2.58	−0.15
	Random	Residual	30.99	<0.001	27.73	34.63
		Intercept (Community)	8.47	0.019	3.68	19.51

The fixed-effect and random-effect/covariance estimates (b) for each parameter are given with the corresponding t-test P values and 95% confidence intervals. b indicates whether a relationship between the parameter and IgG1 galactosylation is positive or negative.
