# Supplementary material for: IgG1 Fc N-glycan galactosylation as a biomarker for immune activation
Source: Sci Rep. 2016 Jun 16;6:28207. doi: 10.1038/srep28207 (PMC4910062; doi:10.1038/srep28207)
Supplement: Supplementary Information [file srep28207-s1.pdf]

# IgG1 Fc N-glycan galactosylation as a biomarker for immune activation

Sanne E. de Jong[1], Maurice H.J. Selman[2], Ayola A. Adegnika[1,3,4], Abena S. Amoah[1,5], Elly van Riet[1], Yvonne C.M. Kruize[1], John G. Raynes[6], Alejandro Rodriguez[7], Daniel Boakye[5], Erika von Mutius[8,9], André C. Knulst[10], Jon Genuneit[11], Philip J. Cooper[7,12], Cornelis H. Hokke[13], Manfred Wuhrer[2], Maria Yazdanbakhsh[1,*]

[1]Leiden Immunoparasitology Group, Department of Parasitology, Leiden University Medical Center, Leiden, the Netherlands, [2]Center for Proteomics and Metabolomics, Leiden University Medical Center, Leiden, the Netherlands, [3]Center of Medical Research Lambaréné (CERMEL), Lambaréné, Gabon, [4]Institute for Tropical Medicine, University of Tübingen, Tübingen, Germany, [5]Department of Parasitology, Noguchi Memorial Institute for Medical Research, University of Ghana, Legon, Accra, Ghana, [6]Department of Immunology and Infection, London School of Hygiene and Tropical Medicine, London, United Kingdom, [7]Instituto de Microbiologia, Universidad San Francisco de Quito, Quito, Ecuador, [8]Dr von Hauner Children's Hospital, Ludwig Maximilian University, Munich, Germany, [9]Comprehensive Pneumology Center Munich (CPC-M), Member of German Center for Lung Research (DZL), Munich, Germany, [10]Department of Dermatology/Allergology, University Medical Center Utrecht, Utrecht, the Netherlands, [11]Institute of Epidemiology and Medical Biometry, Ulm University, Ulm, Germany, [12]Institute of Infection and Immunity, St George's University of London, London, United Kingdom, [13]Leiden Parasite Glycobiology Group, Department of Parasitology, Leiden University Medical Center, Leiden, the Netherlands.

*Address correspondence to M.Y. (m.yazdanbakhsh@lumc.nl).

# Supplementary information

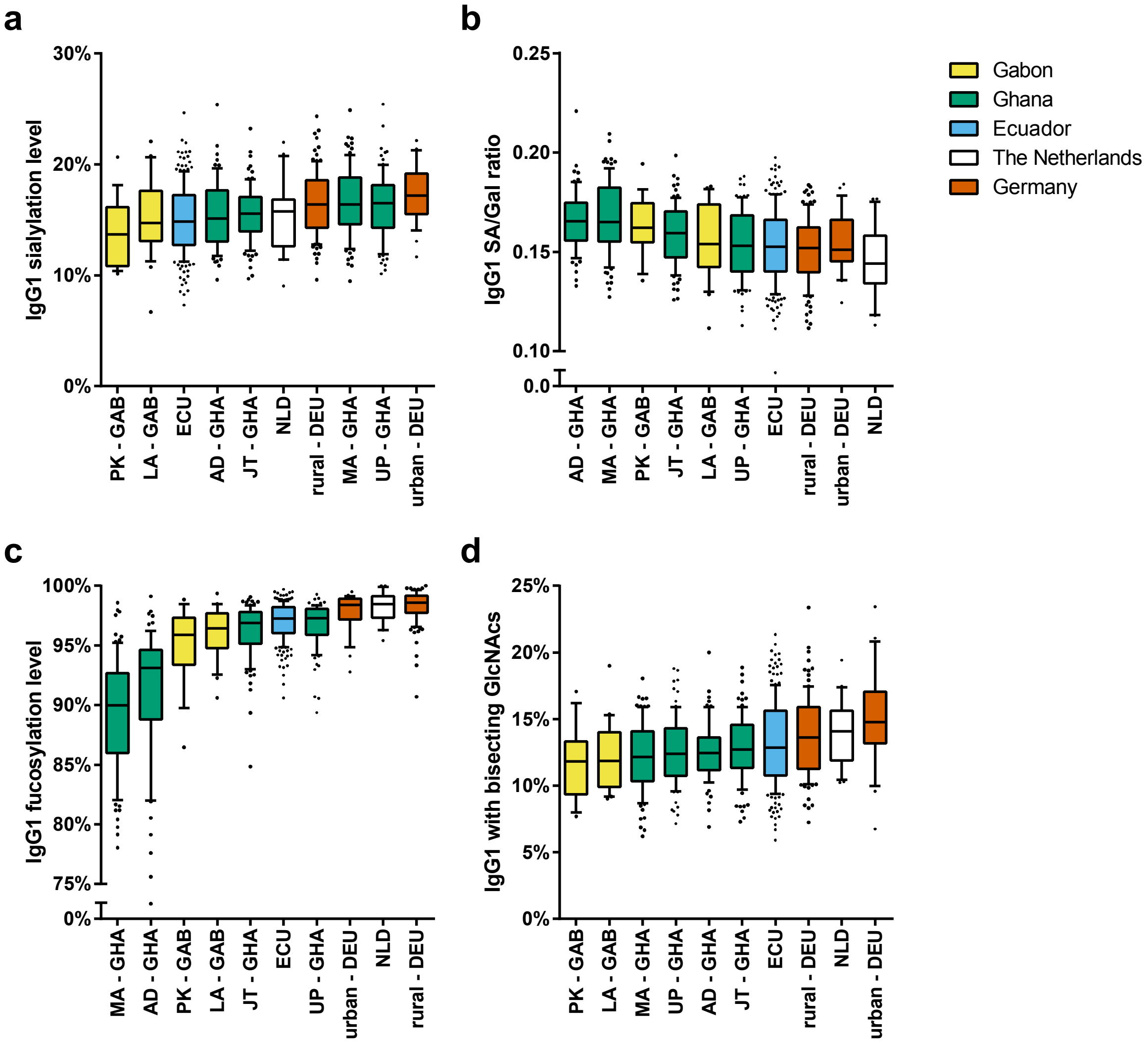


**Supplementary Figure S1.** a) Sialylation, b) sialic acids per galactose, c) fucosylation and d) abundance of bisecting GlcNAcs of IgG1 compared between different communities and countries. Boxplots with 10-90% whiskers.

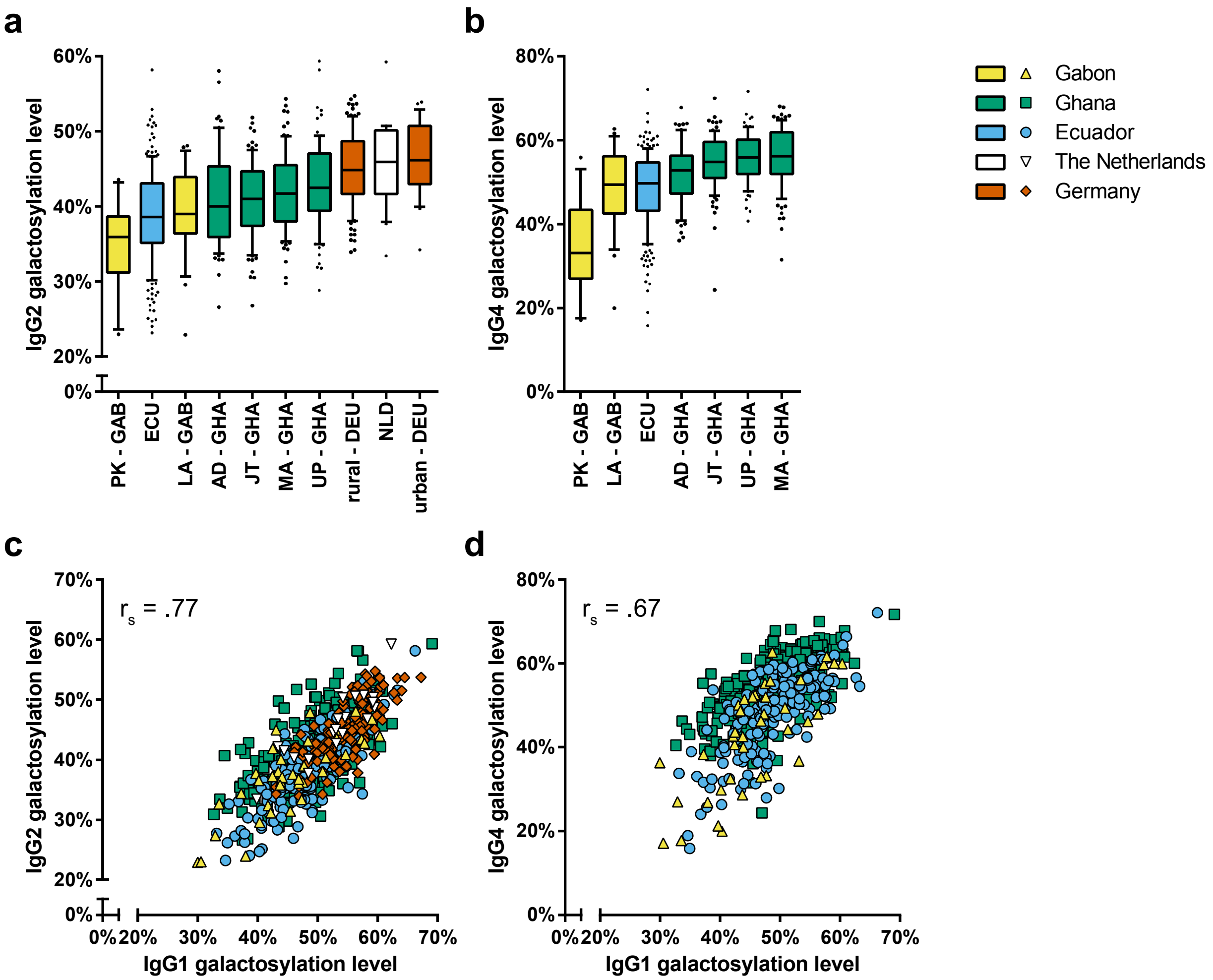


**Supplementary Figure S2.** a) IgG2 galactosylation compared between various communities and countries. b) IgG4 galactosylation compared between populations. Germany and the Netherlands were left out of the analysis, as the IgG4 levels of many children was below detection limit. c) Spearman's correlation between IgG1 galactosylation and IgG2 galactosylation (n=700). d) Spearman's correlation between IgG1 galactosylation and IgG4 galactosylation (n=555). Boxplots have 10-90% whiskers. Spearman's rho correlation coefficient $r_s$ is shown, with $P < .001$.

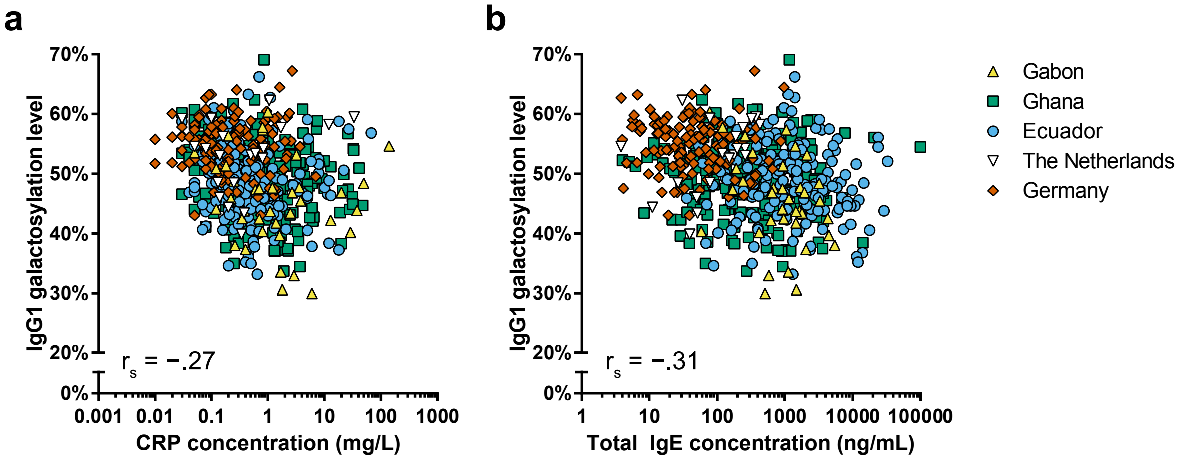


**Supplementary Figure S3.** Spearman's correlation between IgG1 galactosylation and a) CRP levels and b) total IgE levels. Spearman's rho correlation coefficient $r_s$ is shown, with $P < .001$ (n=700).

**Supplementary Table S1.** Comparison of IgG1 galactosylation between all countries, between all communities and between communities within a country with the non-parametric Kruskal-Wallis H test and Mann Whitney U test. IgG1 galactosylation differed significantly between the various countries and communities. When comparing communities within one country only, the Gabonese communities differed significantly from each other, as did the Ghanaian communities, while the Ecuadorian and German communities did not. Post-hoc testing results are shown in Supplementary Table S3 and Table S4.

| Comparison of IgG1 galactosylation | Total number of children | Test statistics | P value |
|---|---|---|---|
| Between the 5 countries | 700 | H(4) = 112.08 | <.001 |
| Between the 16 communities of all countries | 700 | H(15) = 163.59 | <.001 |
| Between the 2 Gabonese communities | 39 | U = 91.00, z = −2.66 | .007 |
| Between the 4 Ghanaian communities | 323 | H(3) = 39.50 | <.001 |
| Between the 7 Ecuadorian communities | 193 | H(6) = 10.24 | .111 |
| Between the 2 healthy German communities | 125 | U = 988.00, z = −1.62 | .107 |

**Supplementary Table S2.** P values resulting from Dunn-Bonferroni post-hoc tests for comparison between all countries as in Supplementary Table S2. IgG1 galactosylation differed significantly between Gabon and Ghana, Gabon and Germany, Gabon and the Netherlands, Ghana and Germany, Ecuador and Germany, and Ecuador and the Netherlands.

| Country | P value post-hoc tests for comparisons between countries | | | | |
|---|---|---|---|---|---|
| | Gabon | Ghana | Ecuador | Germany | The Netherlands |
| Gabon | | .034 | .276 | <.001 | .001 |
| Ghana | .034 | | 1.000 | <.001 | .099 |
| Ecuador | .276 | 1.000 | | <.001 | .027 |
| Germany | <.001 | <.001 | <.001 | | 1.000 |
| The Netherlands | .001 | .099 | .027 | 1.000 | |

**Table S3.** P values resulting from Dunn-Bonferroni post-hoc tests for comparison of communities within Ghana as in Supplementary Table S3. IgG1 galactosylation differs significantly between the Ghanaian communities AD, MA and JT as compared to UP.

| Community | P value post-hoc tests for comparison between communities | | | |
|---|---|---|---|---|
| | GHA, AD | GHA, MA | GHA, JT | GHA, UP |
| GHA, AD | | .052 | .222 | <.001 |
| GHA, MA | .052 | | 1.000 | .001 |
| GHA, JT | .222 | 1.000 | | <.001 |
| GHA, UP | <.001 | .001 | <.001 | |

**Supplementary Table S4.** Spearman's correlation coefficient $r_s$ between various IgG1 glycan modifications, with accompanying P values.

| Modification | Galactosylation | | Sialylation | | SA/Gal | | Fucosylation | | Bisecting GlcNAc | |
|---|---|---|---|---|---|---|---|---|---|---|
| | $r_s$ | P value | $r_s$ | P value | $r_s$ | P value | $r_s$ | P value | $r_s$ | P value |
| Galactosylation | | | .81 | <.001 | .29 | <.001 | .16 | <.001 | −.03 | .497 |
| Sialylation | .81 | <.001 | | | .77 | <.001 | −.03 | .500 | −.07 | .051 |
| SA/Gal | .29 | <.001 | .77 | <.001 | | | −.21 | <.001 | −.10 | .012 |
| Fucosylation | .16 | <.001 | −.03 | .500 | −.21 | <.001 | | | .12 | .002 |
| Bisecting GlcNAc | −.03 | .497 | −.07 | .051 | −.10 | .012 | .12 | .002 | | |

**Supplementary Table S5.** Spearman's correlation coefficient $r_s$ of various glycan modifications between IgG1, IgG2 and IgG4 subclasses, with accompanying P values. Germany and the Netherlands were left out of the analysis with IgG4, as the IgG4 levels of many children was below detection limit.

| Modification | IgG1 with IgG2 | | IgG1 with IgG 4 | | IgG2 with IgG 4 | |
|---|---|---|---|---|---|---|
| | $r_s$ | P value | $r_s$ | P value | $r_s$ | P value |
| Galactosylation | .77 | <.001 | .67 | <.001 | .63 | <.001 |
| Sialylation | .75 | <.001 | .75 | <.001 | .71 | <.001 |
| SA/Gal | .80 | <.001 | .75 | <.001 | .62 | <.001 |
| Fucosylation | .36 | <.001 | | | | |
| Bisecting GlcNAc | .78 | <.001 | .74 | <.001 | .66 | <.001 |

**Supplementary Table S6.** Definitions and descriptive characteristics of the infrastructure, socioeconomic and lifestyle indicators of the Ecuadorian population, as determined by Rodriquez *et al*[12].

| | Indicators | Definition | Categories |
|---|---|---|---|
| Infrastructure | Administrative grade | Political/administrative division | Towns, parish |
| | Transport access | Type of access used to arrive at communities | River, road |
| | Electrical grid | Presence of a connection to the electrical grid | No, yes |
| | Piped water system | Presence of a piped water system (untreated water only) | No, yes |
| | Telephone system | Access to the national telephone network | No, yes |
| | Health centre | Presence of a health centre | No, yes |
| | Pharmacy | Presence of a pharmacy | No, yes |
| | Secondary school | Presence of secondary schools | No, yes |
| | Shops | Number of shops (commercial infrastructure) | |
| socioeconomics | Father's education | % of households in which the father has a secondary education | |
| | Mother's education | % of households in which the mother has a secondary education | |
| | Household income | % of households with an income ≥ US$150/month | |
| | Access to electricity | % of households with electricity (electrical grid or generator) | |
| | Material goods | % of households with (all of) refrigerator, TV and stereo system | |
| | Cement house | % of households with cement walls | |
| | Gas for cooking | % of households that use propane gas for cooking | |
| | Motor vehicles | % of households with motor vehicles (boat or car) | |
| | Crowded household | % of households that are crowded (>3 persons per bedroom) | |
| Lifestyle | Farming activities | % of households that work in agricultural activities | |
| | Cat in house | % of households that have a cat living inside the house | |
| | Dog in house | % of households that have a dog living inside the house | |
| | TV in house | % of households that have a television | |
| | TV viewing | % of study children who watch television >1 h daily | |
| | Hamburger consumption | % of study children who consume ≥ 1 hamburger per month | |
| | Fizzy drinks consumption | % of study children who consume fizzy drinks daily | |
| | Physical exercise | % of study population with daily physical activity (not sedentary) | |
| | Migration | % of study children who has lived for ≥3 months outside the study area in the past | |
| | STH infection rate | % of study population with intestinal helminth *Ascaris lumbricoides, Trichuris trichiura, Strongyloides stercoralis* | |

**Supplementary Table S7.** Theoretical m/z values of human IgG Fc glycopeptides detected.

| Glycan species[a] | IgG1 P01857[b] $E_{293}EQYNSTYR_{301}$[c] | | IgG2 P01859[b] $E_{293}EQFNSTFR_{301}$[c] | | IgG4 P018561[b] $E_{293}EQFNSTYR_{301}$[c] | |
|---|---|---|---|---|---|---|
| | $[M + 2H]^{2+}$ | $[M + 3H]^{3+}$ | $[M + 2H]^{2+}$ | $[M + 3H]^{3+}$ | $[M + 2H]^{2+}$ | $[M + 3H]^{3+}$ |
| No | 595.260 | 397.176 | 579.265 | 386.513 | 587.262 | 391.844 |
| G0F | 1317.527 | 878.687 | 1301.532 | 868.024 | 1309.529 | 873.356[d1] |
| G1F | 1398.553 | 932.705 | 1382.558 | 922.042 | 1390.556 | 927.373[d2] |
| G2F | 1479.580 | 986.722 | 1463.585 | 976.059 | 1471.582 | 981.391 |
| G0FN | 1419.067 | 946.380 | 1403.072 | 935.717 | 1411.069 | 941.049[d3] |
| G1FN | 1500.093 | 1000.398 | 1484.098 | 989.735 | 1492.096 | 995.066[d4] |
| G2FN | 1581.119 | 1054.416 | 1565.124 | 1043.752 | 1573.122 | 1049.084 |
| G1FS | 1544.101 | 1029.737 | 1528.106 | 1019.073 | 1536.104 | 1024.405[d5] |
| G2FS | 1625.127 | 1083.754 | 1609.132 | 1073.091 | 1617.130 | 1078.423 |
| G1FNS | 1645.641 | 1097.430 | 1629.646 | 1086.766 | 1637.643 | 1092.098 |
| G2FNS | 1726.667 | 1151.447 | 1710.672 | 1140.784 | 1718.670 | 1146.116 |
| G0 | 1244.498 | 830.001 | 1228.503 | 819.338 | - | - |
| G1 | 1325.524 | 884.019 | 1309.529 | 873.356[d1] | - | - |
| G2 | 1406.551 | 938.036 | 1390.556 | 927.373[d2] | - | - |
| G0N | 1346.038 | 897.694 | 1330.043 | 887.031 | - | - |
| G1N | 1427.064 | 951.712 | 1411.069 | 941.049[d3] | - | - |
| G2N | 1508.090 | 1005.730 | 1492.096 | 995.066[d4] | - | - |
| G1S | 1471.072 | 981.051 | 1455.077 | 970.387 | - | - |
| G2S | 1552.098 | 1035.068 | 1536.104 | 1024.405[d5] | - | - |
| G1NS | 1572.612 | 1048.744 | 1556.617 | 1038.081 | - | - |
| G2NS | 1653.638 | 1102.761 | 1637.643 | 1092.098 | - | - |

[a]Glycan structural features are given in terms of number of galactoses (G0, G1, G2), fucose (F), bisecting *N*-acetylglucosamine (N), and *N*-acetylneuraminic acid (S). [b]SwissProt entry number. [c]Tryptic IgG glycopeptide sequence. [d]Isomeric glycopeptide species of IgG2 and IgG4.
